# Fear of Falling After Total Knee Replacement: A Saudi Experience

**DOI:** 10.3390/clinpract15080146

**Published:** 2025-08-06

**Authors:** Turki Aljuhani, Jayachandran Vetrayan, Mohammed A. Alfayez, Saleh A. Alshehri, Mohmad H. Alsabani, Lafi H. Olayan, Fahdah A. Aljamaan, Abdulaziz O. Alharbi

**Affiliations:** 1 Department of Occupational Therapy, College of Applied Medical Sciences, King Saud bin Abdulaziz University for Health Sciences, Riyadh 11481, Saudi Arabia; vetrayanj@ksau-hs.edu.sa (J.V.); alfayez10390@ksau-hs.edu.sa (M.A.A.);; 2 King Abdullah International Medical Research Center, Riyadh 11481, Saudi Arabia; sabanim@ksau-hs.edu.sa (M.H.A.); olayanl@ksau-hs.edu.sa (L.H.O.); aljamaanfa1@mngha.med.sa (F.A.A.); harbia3@mngha.med.sa (A.O.A.); 3 Anesthesia Technology Department, College of Applied Medical Sciences, King Saud bin Abdulaziz University for Health Sciences, Riyadh 11481, Saudi Arabia; 4 Department of Rehabilitation Services, King Abdulaziz Medical City, Riyadh 11481, Saudi Arabia

**Keywords:** fear of falling, total knee arthroplasty, quality of life, rehabilitation

## Abstract

**Background**: Fear of falling (FOF) is a significant concern among older adults, especially after total knee arthroplasty (TKA). FOF can limit daily activities, reduce quality of life, and hinder recovery. This study aimed to investigate the prevalence, severity, and impacts of FOF in patients undergoing TKA and identify factors contributing to increased FOF. **Methods**: A prospective observational study was conducted at King Abdulaziz Medical City in Riyadh, Saudi Arabia, from April 2024 to December 2024. This study included 52 participants aged 20 to 75 years who had undergone primary TKA. Data were collected at two time points: after TKA and at three months post-surgery. The Short Falls Efficacy Scale-International (SFES-I) was used to assess the severity of FOF, and the Short Form 36 (SF-36) was used to measure the quality of life. Descriptive statistics, *t*-tests, and logistic regression were used for analysis. **Results**: This study included 52 participants (mean age: 63.77 ± 6.65 years; 82.7% female). Post-TKA, all participants exhibited high FOF (mean SFES-I score: 56.75 ± 8.30). After three months, the mean SFES-I score decreased significantly to 49.04 ± 12.45 (*t* = 4.408, *p* < 0.05). Post-TKA, SF-36 showed significant improvements in the physical function, role of physical limitations, bodily pain, vitality, social function, role of emotional limitations, and mental health subdomains. Bilateral total knee arthroplasty, body mass index, and some SF-36 subcomponents—such as general health, vitality, and role of emotional limitations—were identified as factors leading to increased FOF. **Conclusions**: FOF remains prevalent and severe in TKA patients, even at three months post-surgery, affecting rehabilitation outcomes. Early identification and tailored interventions for FOF should be considered essential components of comprehensive TKA recovery programs.

## 1. Introduction

Falls are a major concern in older adults, being a leading cause of injury in community-dwelling older adults [1]. A fall is defined as an incident that results in a person coming to rest unintentionally on the ground, floor, or other lower level [2]. Falls can lead to injuries that may be fatal or non-fatal, with the majority being non-fatal. It is estimated that one in three adults over the age of 65 falls each year [3]. The prevalence of falls among community-dwelling older adults in Saudi Arabia ranges from 34 to 57% [4]. Other, more recent studies have reported the prevalence of falls to be 49.9% and 47.4% [5,6]. Falls can cause physical harm, reduce psychological function, cause loss of independence, and impair physical activities [7]. Thus, falls may have a major impact on the affected person, their family/caregiver, and society in general.

Multiple risk factors have been found to increase the risk of falls among adults. Osteoarthritis (OA) is an important risk factor of falls, which is a widely prevalent age-associated joint condition. Half of all OA patients and 65% of female OA patients reported falling within the past year [8,9,10]. While there are new promising therapeutic interventions for OA to reduce pain and improve function, such as carboxymethyl chitosan [11,12], there is still a lack of effective treatments to reverse the progression of OA [13]. Total knee arthroplasty (TKA) is a common surgical intervention that can help with knee OA. The aim of TKA is to relieve pain, re-establish locomotor functions, and improve quality of life [14,15]. However, multiple studies have reported that patients with knee OA who undergo TKA are at increased risk of falls, with the rate of falls being reported as high as 7% to 40% post-TKA [16,17,18,19]. While it is still unclear whether TKA affects the patients’ balance or fall rates, patients with TKA usually report a significant loss of proprioceptive acuity and balance control, which is frequently brought on by a lack of confidence [20].

Fear of falling (FOF) is a sensation of concern about the danger of falling, which can be sufficient to hinder a person’s involvement in daily activities [21]. The relationship between FOF and TKA has been examined in multiple studies. For example, one study found that 56.5% of TKA patients reported fear of falling [22]. The rate of FOF tends to decrease post-operatively, when compared to pre-operative levels [7,23]. Rehabilitation and exercise interventions can explain the decrease in FOF. In fact, rehabilitation and exercise interventions post-TKA are linked to reduction in pain, improved function, improved self-efficacy, and a decrease in FOF [24,25].

There is a lack of literature in Saudi Arabia regarding FOF in general and specifically for patients with TKA. Almarwani [26] reported a high level of FOF in the majority of community-dwelling older women, regardless of their history of falling. In addition, an association between FOF and performance in the Sit-to-Stand Test was found in community-dwelling older adults in Saudi Arabia [27]. However, to our knowledge, no previous study has examined the rate and severity of FOF in patients with TKA in Saudi Arabia.

This study has two aims: (1) to investigate the severity and impact of FOF in patients undergoing TKA using the Short Falls Efficacy Scale International (SFES-I) and Short Form 36 (SF-36) after TKA and at three months post-surgery (3 months after rehabilitation); and (2) to investigate factors that may increase FOF in TKA patients. We hypothesize that (a) patients will present an increased FOF and greater severity post-TKA; and (b) a decrease in FOF will be noted post-rehabilitation.

## 2. Materials and Methods

### 2.1. Study Design

This is a prospective observational study which was conducted to explore the feasibility of assessing FOF and its determinants in patients undergoing TKA who were admitted to the hospital and attended post-operative rehabilitation sessions. Data was collected at two time points: (1) post-TKA operation, and (2) three months post-operation. Three months measure was selected as this time point would usually be the end of the rehabilitation sessions for TKA patients. A non-probability convenience sampling method was used to recruit adults aged 20 to 75 years who met the inclusion criteria. All participants were informed about the study and provided informed consent before completing the self-reported assessments. All participants were recruited from the surgical waiting list and were scheduled to have the TKA surgery within the study period.

**Figure 1:**
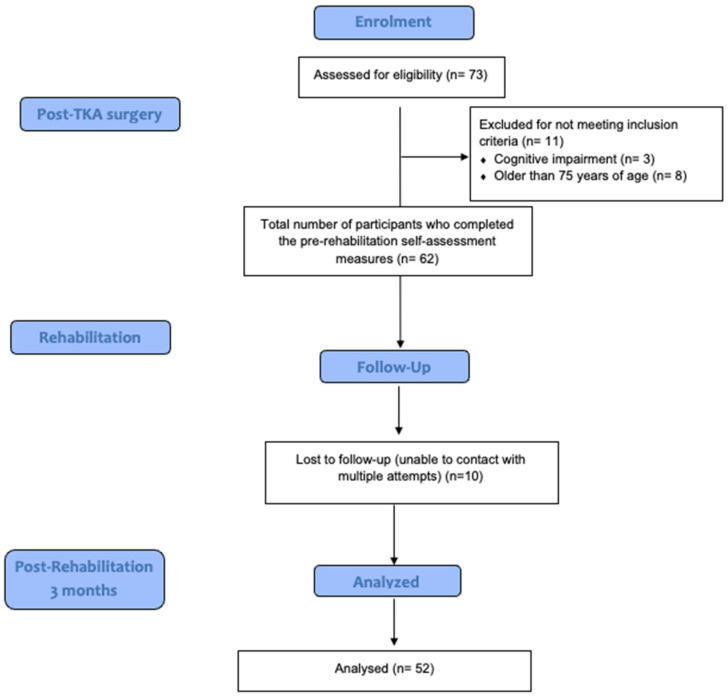
Flowchart of the recruitment process and timeline of assessments.

The rehabilitation department at King Abdulaziz Medical City in Riyadh (KAMC-R) was the study site. In particular, the study included patients who were scheduled for TKA at KAMC-R between April 2024 and March 2025. Patients between the ages of 20 and 75 years and who had TKA as the primary reason for admission and received rehabilitation services at the center were included in the study. Patients with cognitive impairment and a history of two or more falls within the past year were excluded from the study. Participants’ co-morbidities, demographic information, and medical history were extracted from their KAMC-R medical records (BestCare, ezCareTech, Seoul, South Korea). The required sample size was calculated using the G*Power software (version 3.1) using the Wilcoxon signed-rank test for paired samples. Assuming a moderate effect size (Cohen’s dz = 0.4), a significance level (α) of 0.05, and a power of 0.8, the required sample size was calculated to be *n* = 54. Accounting for an anticipated dropout rate of 20%, the final target sample size was increased to 64 [28]. Figure 1 shows the participant recruitment process and the timeline of the assessments. Our study’s final sample size was slightly lower (*n* = 52) than the calculated sample size, which was probably due to the high follow-up dropout rate, as well as our inclusion criterion of only recruiting participants of age ≤ 75.

### 2.2. Outcome Measures

#### 2.2.1. Demographic Data

The first section collected sociodemographic characteristics and health conditions, including age, gender, present comorbidities, history of falls, side/s of the surgery, and body mass index (BMI). BMI was calculated by dividing the body weight in kilograms by height in meters squared (kg/m^2^). Classification followed the World Health Organization categories for adults: a BMI of ≥30.0 kg/m^2^ was classified as obese, 25.0–30.0 kg/m^2^ as overweight, 18.5–24.9 kg/m^2^ as healthy weight, and <18.5 kg/m^2^ as underweight.

#### 2.2.2. Short Fall Efficacy Scale-International (SFES-I)

The first primary outcome variable was the Short Fall Efficacy Scale-International (SFES-I), which was used to assess the fear of falling in patients after surgery. It categorizes the severity of fall-related anxiety into low, moderate, and high levels based on the responses to a series of questions related to daily activities [29,30]. The SFES-I measures a person’s level of anxiety about falling using different instrumental activities of daily living (IADLs) [30]. The short FES-I consist of seven items, each rated on a four-point scale (1 = not at all worried, 2 = a little worried, 3 = somewhat worried, 4 = very worried) [29]. The obtained scores are then categorized as follows: 7–8 indicates low fear of falling, 9–13 indicates moderate fear of falling, and 14–28 indicates high fear of falling [29]. The Arabic version of the SFES-I showed a high percentage of item agreements, ranging from 88 to 93%, and was positively correlated with the Timed Up and Go test and negatively correlated with gait speed and balance [31]. Thus, the Arabic SFES-I can be considered a valid and reliable measure of fear of falling [31,32].

#### 2.2.3. Short Form Health Survey-36

The second primary outcome measure was the Short Form Health Survey-36 (SF-36) score. The SF-36 measures the general quality of life (QoL) across 8 domains, including 10 items measuring physical function (PF), 4 items measuring the role of physical limitations (RP), 2 items measuring bodily pain (BP), 2 items measuring social functioning (SF), 3 items measuring the role of emotional limitations (RE), 5 items measuring general health (GH), 4 items measuring vitality (VT), and 5 items measuring mental health (MH) [33]. Each item has a score ranging from 0 to 100, with higher scores suggesting better health. The Arabic version of the SF-36 has excellent inter-rater reliability and good construct validity [34].

Both self-reported assessments were self-administered and were completed at two time points: 1. post-TKA (pre-rehabilitation), and 2. at three months post-TKA (post-rehabilitation). The selected time points were chosen as they allowed the rehabilitation team more direct access to the participants. Pre-surgical assessments (prior to TKA) were not conducted as it was not certain that all patients would utilize the rehabilitation services within the center, thus making it difficult to track the participants.

### 2.3. Statistical Analysis

After data collection, the responses were entered into Microsoft Excel for initial data cleaning. The data were carefully checked for completeness and consistency. After cleaning, the data were exported to IBM SPSS statistics (version 27.0.0.1) for further statistical analysis. Descriptive statistics, including means and standard deviations, were calculated to summarize the demographic characteristics of the study population and the outcome variables.

To assess the differences in SFES-I and SF-36 scores between groups, paired *t*-tests were performed. The Pearson correlation test was employed to examine the relationship between post-SFES-I scores and the subdomains of the SF-36 quality of life questionnaires. A logistic regression model was used to identify factors associated with an increased fear of falling after TKA, adjusting for potential confounders. Statistical significance was set at a *p*-value of less than 0.05 for all tests.

### 2.4. Ethical Consideration

This study obtained ethical approval from the institution review board (IRB) of King Abdullah International Medical Research Center (KAIMRC), with a study approval number (IRB/0684/24, date of approval 18 April 2024). All participants were given detailed information about the study and gave written informed consent before enrollment. The Strengthening the Reporting of Observational Studies (STROBE) checklist was followed for the reporting of data and results in this study (Supplementary File S1).

## 3. Results

A total of 52 participants were included in this feasibility study. The mean age of the participants was 63.77 ± 6.65, with more female participants (82.7% vs. 17.3% males). The mean BMI of the participants was 33.6 ± 5.06, most of the participants were classified as obese (78.8% of the total participants), and 50% of the participants had both diabetes and hypertension. There was a higher percentage of TKA performed on the right side (at 55.8%) than on the left side (40.4%), while bilateral surgery was performed in only 3.8% of patients. A total of 59.1% of the participants had a history of falling within the past year (Table 1).

The data in Table 2 show that, after TKA surgery, fear of fall scores on the SFES-I scale were 56.75 ± 8.30 while, after 3 months of rehabilitation, they decreased to 49.04 ± 12.45, with a significant difference between groups (*t* = 4.408; *p* < 0.05). The subjects’ FOF ratings were still high after rehabilitation but were much lower than after surgery.

The paired *t*-test results comparing pre- and post-rehabilitation quality of life scores utilizing the SF-36 indicated a significant difference across various subdomains, including PF (pre: 16.92 ± 17.71; post: 41.25 ± 41.25; *t* = −7.88; *p* = 0.000), RP limitations (pre: 16.83 ± 30.80; post: 38.94 ± 41.54; *t* = −3.31; *p* = 0.002), BP (pre: 32.30 ± 25.17; 54.47 ± 27.92; *t* = −4.87; *p* = 0.000), VT (pre: 50.29 ± 19.26; post: 60.77 ± 24.08; *t* = −2.77; *p* = 0.008), SF (pre: 53.37 ± 26.09; post: 65.84 ± 27.24; *t* = −3.33; *p* = 0.002), RE limitations (pre: 49.36 ± 47.82; post: 64.10 ± 47.70; *t* = −2.12; *p* = 0.038), and MH (pre: 73.30 ± 21.55; post: 80.69 ± 15.94; *t* = −2.59; *p* = 0.012). While the GH scores slightly decreased, the difference did not reach statistical significance (*p* = 0.296) (Table 3).

The correlation analysis examined the variables between the post-rehabilitation FOF and QoL (SF-36) subdomain scores. The results revealed a low negative correlation between the FOF and the GH subdomain of the SF-36 (*r* = 0.26; *p* = 0.057), such that a decrease in general health slightly increased the FOF. Moreover, the other subdomains of the SF-36 presented low correlations but did not reach the significance level of *p* < 0.05 (Table 4).

Linear regression analysis was performed to identify the predictors of FOF from the different demographic variables and outcome measures. The results indicated bilateral TKA as the major predictor of FOF among the participants (*β* = 16.37, *p* = 0.043, Wald chi square 4.10, CI [0.53, 32.21]), which was followed by VT (*β* = 0.28, *p* = 0.010, Wald chi square 6.65, CI [0.07, 0.49]), BMI (*β* = 0.62; *p* = 0.041, Wald chi square 4.17, CI [−1.22, −0.02]), RE (*β* = 0.10; *p* = 0.031 Wald chi square 4.66, CI [−0.19, 0.01]), and GH (*β* = 0.28; *p* = 0.014, Wald chi square 6.03, CI [−0.50, −0.06]); meanwhile, the other variables did not reach the significance level (*p* > 0.05) (Table 5). The liner regression showed a medium effect size of 0.77, indicating a substantial difference between the groups. It illustrates that the predicting variables have a 69% effect on the FOF among the participants [28].

## 4. Discussion

The incidence of falls has shown a substantial rise among the older Saudi Arabian population, with more than 130,000 fall cases reported in 2019 [35]. FOF is a well-known modifiable risk factor for falls which may be problematic for this patient population, especially when physical activities are encouraged during the post-operative recovery period [36]. It has been reported that FOF increases the risk of falling by 12 times post-TKA [37]. Few studies have investigated factors associated with FOF in TKA patients in Saudi Arabia during the immediate post-operative period and 3 months post-rehabilitation. The study examined the severity and impact of FOF in patients post-TKA rehabilitation, its quality of life, and the factors influencing FOF.

Our study showed a high level of concern for FOF during the immediate post-operative period among patients who underwent TKA. A significant reduction in concern for FOF was observed after rehabilitation interventions (i.e., at three months post-surgery), with respect to the SFES-I score, although it was still at a relatively high level. These findings are in line with a previous quasi-experimental study conducted in Turkey that included 70 TKA patients who either underwent usual care or an educational program on fall prevention [38]. The authors reported that, in the control group, a high level of concern for FOF was observed before TKA and at 6–8 weeks and 14–16 weeks after surgery [39]. Patients who attended the educational program before surgery in the same study showed a significant improvement in mean SFES-I scores, with the overall FOF level reduced to a moderate level at 14–16 weeks after surgery. A more recent study estimated the FOF measured using the SFES-I at six months post-operation to be as high as 65% [39]. In another study by Hai-bo Si and colleagues, patients who underwent primary unilateral TKA reported low concern for FOF pre-operatively (mean SFES-I score: 15.89  ±  4.72) [15]. The authors also observed significantly lower scores after post-operative follow-up by one year (mean SFES-I score: 12.40  ±  3.16) and two years (mean SFES-I score:11.24  ±  2.43). Their findings may be attributed to the fact that remarkable healing after TKA starts within 3–4 months after surgery, and optimal healing is achieved at one year post-operatively [40,41]. Post-operative rehabilitation services which include exercise, strength, gait, and balance training have significant positive impacts on mobility and function for patients with fractures [41]. Thus, it would be beneficial to include rehabilitation services even prior to TKA to improve patient outcomes [20]. Furthermore, a qualitative study in Kuwait revealed delayed TKA due to delayed clinical advice and lack of patients education, lead to unrealistic patient expectations and goals [42]. Multiple studies in Saudi Arabia have reported FOF and falls; for example, one study reported the prevalence among older adults as 46.5% [43], while another study reported the prevalence of falls as 49.9% among Saudi adults aged over 60 years old [44].

Our study showed improvements in the overall health-related quality of life measures at three-months after surgery, when compared to immediate post-operative results. Noteworthy improvements were observed in the BP, VT, SF, RE, and MH subscales of the SF-36 self-perceived QoL survey. Other measures—such as PF and RP—showed significant improvements at three months post-surgery, but the mean scores suggested that severe limitations still exist. These findings are consistent with previous research highlighting poor physical functioning post-TKA, which can likely be attributed to various conditions such as pain, the presence of comorbid conditions, and BMI [45,46]. Hence, advanced treatment processes should focus on the physical components in order to increase physical health and improve the quality of life of patients undergoing TKA surgery. Furthermore, our findings suggest the importance of the roles of emotion and mental health in recovery post-TKA. Thus, interventions related to maintaining or improving mental health may help patients in the post-TKA period, possibly further influencing physical function and pain [47,48].

In our study, through the linear regression analysis, factors found to be associated with FOF included bilateral TKA, VT, RE, and GH. BMI and anxiety are important risk factors for FOF, and patients with increased BMI and higher anxiety levels tend to exhibit a higher likelihood of FOF [22]. In the current study, we observed that bilateral TKA, high BMI, lower general health, higher vitality, and low role of emotional limitations were significant risk factors for FOF after three months of rehabilitation. Most of our results were consistent with other studies, including the findings that having bilateral TKA surgery [49], high BMI [22,49,50], lower general health [51,52], higher vitality, and low role of emotional limitations [22,51] as predictors of increased FOF. Furthermore, vitality has been reported to have effects on fatigue and energy levels, and it was shown that a high level of fatigue with a low level of energy predicts increased FOF. Multiple studies have found that fatigue and feeling exhausted contribute to FOF in adults [51,52,53].

In terms of clinical implications, our findings highlight the significance of rehabilitation services in reducing FOF and improving QoL in patients who have undergone TKA surgery. In addition, the results illustrate the need for additional rehabilitation services at the pre-operative stage, in order to mitigate FOF and functional limitations. Patients undergoing bilateral TKA or with high BMI, lower general health, high vitality, and low role of emotional limitations should be screened for FOF both pre- and post-operatively. Finally, the use of fall prevention programs is recommended for all patients with TKA, as psychological interventions—such as Cognitive Behavioral Therapy—alongside rehabilitation can notably reduce FOF [54,55,56]. The strengths of the current study include that it examined the immediate effect of rehabilitation post-TKA and used a self-perceived QoL survey at two points (i.e., pre- and post-rehabilitation after TKA).

However, the study also had several limitations. First, the sample size was small (*n* = 52), which can limit the power of the findings. This reduced sample size can potentially impact the generalizability of our findings. The high number of participants who were lost at follow-up (*n* = 10), greatly impacted the power of our current study. Second, relying on a single center limited the external validity of the findings. In addition, the use of a non-probability convenience sampling method may have introduced selection bias, as patients with high levels of pain or who did not benefit from the rehabilitation services were less likely to participate in the study. Our results showed a significant negative correlation between the SF-36 general health subdomain and the post-rehabilitation measure of FOF (according to SFES-I). This association between general health and FOF may have been coincidental; thus, the role of general health as a significant predictor of FOF should be validated. Another limitation was the lack of information on the participants’ medical and mental health histories, social support networks, and use of medication (including antidepressants), which may have influenced their FOF and QoL. Lastly, the study relied on self-reported measures and did not include objective measures (e.g., the Timed Up and Go test or Dynamic Gait Index), potentially impacting the overall results. Future research should include larger, more diverse samples and adopt longitudinal designs to better understand the impacts of FOF on QoL in patients post-TKA.

Our results suggest the importance of screening patients who will undergo TKA surgery for FOF and starting rehabilitation as soon as possible, in order to enhance their QoL. In addition, the presented results support enhancing the medical team’s awareness of FOF prior to TKA surgery, which may help identify patients with high level of FOF and refer them to rehabilitation services for earlier and tailored interventions.

## 5. Conclusions

This study achieved its objectives by identifying the prevalence and severity of FOF in patients post-TKA and highlighting clinical predictors of increased FOF post-rehabilitation. Although physical function, pain, vitality, social functioning, emotional roles, and mental health showed statistically significant improvements (in terms of SF-36 scores) post-rehabilitation, patients continued to report limitations in their daily activities and persistent physical challenges. Importantly, bilateral TKA, higher BMI, lower general health, higher fatigue, and emotional limitations were identified as significant predictors of increased FOF. These findings highlight the need for tailored pre- and post-operative interventions that address both physical and psychological dimensions of recovery. This study’s findings support the potential benefit of incorporating psychological support into post-operative care, although further research is still required to evaluate its effectiveness on overall quality of life for TKA patients. Early identification and tailored interventions for FOF should be considered an essential component of comprehensive TKA recovery programs in the future.

## Figures and Tables

**Table 1:** Demographic data of the participants.

Variable	N (%)	Mean ± SD	Min	Max
Age	52 (100)	63.77 ± 6.65	52	75
Gender				
Male	9 (17.3)			
Female	43 (82.7)			
BMI (kg/m^2^)		33.67 ± 5.06	19.58	44.66
Healthy weight	4 (7.7)			
Overweight	7 (13.5)			
Obese	41 (78.8)			
History of falls (past year)				
Yes	27 (51.9)			
No	25 (48.1)			
Comorbidities				
None	13 (25)			
Diabetes	4 (7.7)			
Hypertension	9 (17.3)			
Both	26 (50)			
Side of surgery				
Bilateral	2 (3.8)			
Left	21 (40.4)			
Right	29 (55.8)			

**Table 2:** Difference between pre- and post-rehabilitation SFES-I scores.

SFES-I	N	Mean	SD	Mean	Std. Deviation	Std. Error Mean	95% Confidence Interval of the Difference		*t*	df	*p*-Value
							Lower	Upper			
Pre—SFES-I	52	56.75	8.30	7.712	12.615	1.749	4.19	11.22	4.408	51	0.000 *
Post—SFES-I	52	49.04	12.45								

* Statistically significant at *p* < 0.05; SFES-I = Short Falls Efficacy Scale-International.

**Table 3:** Difference between pre- and post-rehabilitation scores in SF-36 subdomains for the study participants.

SF-36	N	Mean	SD	Mean	Std. Deviation	Std. Error Mean	95% Confidence Interval of the Difference		t	df	*p*-Value
							Lower	Upper			
Pre—SF-36 PF	52	16.92	17.71	−24.32	22.25	3.08	−30.52	−18.13	−7.88	51	0.000 *
Post—SF-36 PF	52	41.25	24.15								
Pre—SF-36 RP	52	16.83	30.80	−22.11	48.16	6.67	−35.52	−8.70	−3.31	51	0.002 *
Post—SF-36 RP	52	38.94	41.54								
Pre—SF-36 BP	52	32.30	25.17	−22.16	32.83	4.55	−31.30	−13.02	−4.87	51	0.000 *
Post—SF-36 BP	52	54.47	27.92								
Pre—SF-36 GH	52	74.42	13.27	2.40	16.40	2.27	−2.16	6.97	1.05	51	0.296
Post—SF-36 GH	52	72.02	14.62								
Pre—SF-36 VT	52	50.29	19.26	−10.48	27.31	3.79	−18.09	−2.88	−2.77	51	0.008 *
Post—SF-36 VT	52	60.77	24.08								
Pre—SF-36 SF	52	53.37	26.09	−12.48	27.00	3.75	−19.99	−4.96	−3.33	51	0.002 *
Post—SF-36 SF	52	65.84	27.24								
Pre—SF-36 RE	52	49.36	47.82	−14.75	50.02	6.94	−28.67	−0.82	−2.12	51	0.038 *
Post—SF-36 RE	52	64.10	47.70								
Pre—SF-36 MH	52	73.30	21.55	−7.38	20.54	2.85	−13.10	−1.67	−2.59	51	0.012 *
Post—SF-36 MH	52	80.69	15.94								

* Statistically significant at *p* < 0.05; PF = physical function; RP = role of physical limitations; BP = bodily pain; SF = social function; RE = role of emotional limitations; GH = general health; VT = vitality; MH = mental health.

**Table 4:** Correlations between the SFES-I and SF-36 subdomains.

Variables	Pearson Correlation (*r*)	*p*-Value
Post SF-36-GH & Post SFES-I	−0.265	0.057 *
Post SF-36-PF & Post SFES-I	−0.038	0.788
Post SF-36-RF & Post SFES-I	0.185	0.190
Post SF-36-BP & Post SFES-I	0.117	0.409
Post SF-36-VT & Post SFES-I	0.130	0.357
Post SF-36-SF & Post SFES-I	−0.158	0.262
Post SF-36-RE & Post SFES-I	−0.222	0.113
Post SF-36-MH & Post SFES-I	−0.144	0.310

* Correlation is significant at the 0.05 level (2-tailed); PF = physical function; RP = role of physical limitations; BP = bodily pain; SF = social function; RE = role of emotional limitations; GH = general health; VT = vitality; MH = mental health.

**Table 5:** Regression analysis for predictors of FOF and the quality of life subdomains.

Variables	Beta	SE	LL	UL	Wald Chi Square	df	*p*-Value
		**95% CI**					
Fall	−2.21	2.93	−7.97	3.54	0.57	1	0.451
DM	−8.66	5.77	−19.97	2.64	2.26	1	0.133
HTN	5.91	4.01	−1.96	13.77	2.17	1	0.141
Bilateral TKA	16.37	8.08	0.53	32.21	4.10	1	0.043 *
Age	−0.04	0.24	−0.52	0.42	0.04	1	0.841
Post SF-36-PF	0.05	0.04	−0.09	0.19	0.48	1	0.488
Post SF-36-RP	−0.04	0.05	−0.15	0.07	0.48	1	0.488
Post SF-36-BP	0.10	0.07	−0.04	0.25	0.18	1	0.140
Post SF-36-GH	−0.28	0.11	−0.50	−0.06	6.03	1	0.014 *
Post SF-36-VT	0.28	0.11	0.07	0.49	6.56	1	0.010 *
Post SF-36-SF	−0.05	0.07	−0.19	0.09	0.47	1	0.494
Post SF-36-RE	−0.10	0.05	−0.19	0.01	4.66	1	0.031 *
Post SF-36-MH	−0.09	0.15	−0.39	0.22	0.31	1	0.581
BMI	−0.62	0.31	−1.22	−0.02	4.17	1	0.041 *

Note: * Correlation is significant at the *p* = 0.05 level (2-tailed); DM = diabetes mellitus; HTN = hypertension; TKA = total knee arthroplasty; BMI = body mass index; SE = standard error; LL = lower limit; UL = upper limit; PF = physical function; RP = role of physical limitations; BP = bodily pain; SF = social function; RE = role of emotional limitations; GH = general health; VT = vitality; MH = mental health.

## Data Availability

The datasets used and/or analyzed during the current study are available from the corresponding author on reasonable request.

## Supplementary Materials

The following supporting information can be downloaded at: https://www.mdpi.com/article/10.3390/clinpract15080146/s1, File S1: STROBE Statement—Checklist of items that should be included observational studies.

## Abbreviations

The following abbreviations are used in this manuscript:

OA	Osteoarthritis
TKA	Total Knee Arthroplasty
FOF	Fear of falling
SF-36	Short Form 36
SFES-I	Short Falls Efficacy Scale International
QoL	Quality of Life
PF	physical function
RP	role physical
BP	bodily pain
SF	social functioning
RE	role emotional
GH	general health
VT	vitality
MH	mental health
BMI	body mass index
